# Insect cell lines and baculoviruses as effective biocontrol agents of forest pests

**DOI:** 10.1186/1753-6561-5-S8-P71

**Published:** 2011-11-22

**Authors:** Guido F Caputo, Sardar S Sohi, Sharon V Hooey, Lawrence J Gringorten

**Affiliations:** 1 Natural Resources Canada, Great Lakes Forestry Centre, Sault Ste. Marie, ON P6A2E5 CANADA

## Background

Canada is a nation of trees. Canadian forests cover 50% of Canada's land area and represent 10% of world’s forested area. Canada exports more processed forest products than any other country. It is also a host to a variety of forest insects both native and non native that limit the economic, recreational and wildlife habitat use. Our research focuses on initiating insect cell lines, determining nutritional requirements of cells, and developing low cost media for large scale propagation of insect pathogenic viruses as ecologically sound alternatives to chemical pesticides. Cell lines are used for bioassay and strain selection of viruses and bacterial toxins and for production of foreign gene products using baculovirus- and entomopoxvirus expression vectors. They offer a cleaner, viable alternative to insect larvae for producing viral pesticides.

Since 1969, over 150 continuous cell lines have been produced for forest insect pest research at GLFC. This collection represents one of largest single site repository of frozen insect cell lines in the world. Cell lines developed include tissues of the eastern spruce budworm (*Choristoneura fumiferana*), western spruce budworm (*Choristoneura occidentalis*), forest tent caterpillar (*Malacosoma disstria*), tobacco hornworm (*Manduca sexta*), white-marked tussock moth (*Orgyia leucostigma*), red-headed pine sawfly (*Neodiprion lecontei*), gypsy moth (*Lymantria dispar*), white pine weevel (*Pissodes strobi*), the tarnished plant bug (*Lygus lineolaris*) and the ash and privet borer (*Tylonotus bimaculatus*). These cell lines represent six tissues of origin, namely neonate larvae, pre-pupae, embryos, ovaries, hemocytes, and midgut and four Insect Orders namely Lepidoptera, Hymenoptera, Coleoptera and Hemiptera.

Presently, cell lines developed at GLFC are being used by 41 researchers in 8 Canadian provinces, 42 researchers in 21 US States and 28 researchers in 12 countries worldwide.

## Biological control

Although chemical pesticides are generally more reliable and effective control agents, they are not environmentally acceptable as their negative impact far out weigh their benefits. We have developed methodologies to duplicate the *in vivo* process of the insect by producing both baculovirus phenotypes, the occlusion derived virus (ODV) and the budded virus (BV) *in vitro.* During the *in vivo* infection process hemolymph (BV) is collected and cultures are infected producing both phenotypes. BV, released in the media is used to infect other cells; ODV produced in the nuclei to infect other insects.

## Lawn assay

Trees produce numerous compounds with toxic and growth regulating properties to protect themselves against insect attack. We have developed a rapid *in vitro* agarose lawn assay for testing the toxicity of freeze-dried ethanolic leaf extracts and secondary compounds. Foliage from sugar maple, trembling aspen and mulberry was assayed for possible insecticidal properties against cells from two lepidopteran defoliators, spruce budworm and fall armyworm. Cells were suspended in buffered agarose and spread in a petri dish. Leaf extracts solubilized in 50-70% DMSO were applied directly to the cells. Trypan blue staining was used as an indicator of toxicity. Quantitative comparisons were determined by threshold doses that elicited positive responses. Toxicity and validity of the assay were further confirmed by the presence of membrane disruption and cellular lysis. The activity of trembling aspen was markedly enhanced when the pH was raised from 7 to 10.5, a level similar to that of the larval midgut, but the effect was largely abolished in the presence of gut juice. Analysis of a crude mulberry extract treated with neat gut juice suggested that most of the active material in the lepidopteran leaf diet is either insoluble or precipitated in the larval midgut, while the activity of any solubilized material is suppressed through interaction with gut-juice proteins.

## Molecular entomology using insect cell lines

A spruce budworm midgut cell line, FPMI-CF-203 has been shown to respond to the molting hormone ecdysone, juvenile hormone and other chemicals. Not only was gene expression stimulated in these treated cells, but they allowed for the analysis of house keeping genes or molting gene promoters as well as the study of cell signaling pathways such as protein kinase C and its targets, steroid hormone and molting gene expression. In addition, GFP tag fused target proteins were readily visible in single living CF-203 cells. They were permissive for the production of active foreign gene proteins using recombinant baculoviruses vector systems. We have observed that while RNA interference (RNAi) technology for the study of gene function does not work well *in vivo* within the whole insect, CF-203 cells are an invaluable *in vitro* tool for this function.

## Challenges within insect tissue culture discipline

Developing insect cell lines suitable for a specific application is a very slow and challenging process. Primary cultures started may or may not develop into cell lines. Those that do might take months and the resulting cells may not be suitable for a desired function. Of the 4-6 M insect species, we currently have 800+ cell lines from 100 species. There are presently no cell lines from exotic or invasive species. Once established, insect cells, like insects, must be fed to be kept alive. Suitable culture medium for the growth of tissues of different insects is currently not available. Large scale production of insect viruses would require the media to be optimal for cell growth as well as for replication of the virus or other control agents. Each cell line/virus combination requires its own unique media composition. Two pressing concerns requiring immediate attention are cross and misidentification contamination of existing cell lines and retiring of key researchers. With few insect cell lines in public collections, private collections are often inaccessible after the principal investigator leaves. Retiring scientist equals lost insect cell cultures.

## Future requirements to achieve “cultural immortalization”

More dedicated researchers developing new lines from different species, routine and aggressive identification, characterization and verification of cells lines employing new molecular techniques and maximizing the potential of insect cells as viable commercial ventures are needed.

